# Ex Vivo Characterization of Peritoneal Macrophages from Novel ABCA1-LSL and ABCG1-LSL Mice for Macrophage-Specific ABC-Transporter Overexpression

**DOI:** 10.3390/biology14081073

**Published:** 2025-08-18

**Authors:** Jing Echesabal-Chen, Lawrence Fernando, Ally Brawner, Achala Pokhrel, Kun Huang, Rhonda Reigers Powell, Terri Bruce, Jan Guz, Fu-Lei Tang, Alexander Awgulewitsch, Alexis Stamatikos

**Affiliations:** 1 Department of Food, Nutrition, and Packaging Sciences, Clemson University, Clemson, SC 29634, USA; jchen11@clemson.edu (J.E.-C.); brawner@g.clemson.edu (A.B.); achalap@clemson.edu (A.P.); kunh@g.clemson.edu (K.H.); 2 Clemson Light Imaging Facility, Clemson University, Clemson, SC 29634, USA; rhondar@clemson.edu (R.R.P.); terri@clemson.edu (T.B.); 3 Transgenic & Genome Editing Core, Medical University of South Carolina, Charleston, SC 29425, USA; guzj@musc.edu (J.G.); fulei@musc.edu (F.-L.T.); awgulewa@musc.edu (A.A.)

**Keywords:** cell culture, Coronary Artery Disease, Cre-Lox, ischemic stroke, Monocyte, Peripheral Arterial Disease, reverse cholesterol transport, tamoxifen

## Abstract

We aimed to characterize peritoneal macrophages from two novel mouse models that enable macrophage-specific overexpression of ABCA1 and ABCG1 via Cre recombinase. Since ABCA1/ABCG1 expression in macrophages is acknowledged to be anti-atherogenic, overexpression of these two transporters may result in a potent atheroprotective effect. However, there are no current animal models that permit overexpression of ABCA1/ABCG1 to precisely occur in macrophages. The purpose of this work was to use ex vivo strategies to characterize the peritoneal macrophages from two novel mouse models: ABCA1-LSL mice and ABCG1-LSL mice. Overexpression of ABCA1/ABCG1 in peritoneal macrophages is controlled by Cre recombinase, due to the LoxP-Stop-LoxP (LSL) system regulating ABC-transporter overexpression. In this study, we first characterized cells isolated from the peritoneal cavities of mice via immunofluorescent staining with macrophage markers CD11b and F4/80 to confirm that we were successful with peritoneal macrophage isolation. We next isolated peritoneal macrophages from ABCA1-LSL mice and ABCG1-LSL mice, and then we incubated these cells with gesicle particles containing Cre recombinase to measure ABC-transporter expression via immunoblotting and measure apoAI/HDL-mediated efflux using cholesterol efflux assays. In our experiments, we observed increases in ABCA1 protein expression and apoAI-mediated cholesterol efflux within ABCA1-LSL peritoneal macrophages, and increased ABCG1 protein expression and HDL-mediated cholesterol efflux in ABCG1-LSL peritoneal macrophages, when compared to corresponding control peritoneal macrophage groups. In conclusion, this report shows that ABCA1-LSL mice and ABCG1-LSL mice may possibly be utilized to cross with macrophage-specific Cre mice to study ABC-transporter overexpression precisely in macrophages, thereby providing valuable tools to dissect the impact of macrophage-specific ABC-transporter overexpression on atherogenesis.

## Simple Summary

Heart disease and stroke are two major causes of death worldwide. Both conditions are caused by cholesterol accumulating in arteries. Two transporters, ABCA1 and ABCG1, may protect against heart disease and stroke by removing excess cholesterol from artery cells. A major cell type in arteries that accumulates cholesterol is macrophages, and it is thought that increasing ABCA1/ABCG1 expression specifically in macrophages within arteries may protect against heart disease and stroke. However, there are no current animal models able to overexpress ABCA1/ABCG1 specifically in macrophages to directly test this. In this work, we generated mice capable of overexpressing ABCA1/ABCG1 when a protein known as Cre recombinase activates overexpression. When we isolated and cultured macrophages from these mice and then introduced Cre recombinase to these cultured cells, we observed an increase in ABCA1 and ABCG1 expression in these macrophages when compared to corresponding control macrophages not exposed to Cre recombinase. Furthermore, this increase in ABCA1/ABCG1 expression also increased the removal of cholesterol from these cells. Our findings show that we have created new mice that may potentially be utilized to cross with various Cre-driver mice to overexpress ABCA1/ABCG1 in macrophages or other cell types and tissues.

## 1. Introduction

Atherosclerosis is the formation of lipids within the arterial wall, resulting in atherosclerotic lesions [1]. Extensive atherosclerosis can lead to atherosclerotic cardiovascular disease, which is the leading cause of mortality worldwide [2]. Indeed, atherosclerotic cardiovascular disease leads to approximately 50% of all deaths in Western nations [3]. The clinical characteristics of atherosclerosis include angina pectoris, dizziness/pre-syncope, claudication, weakness/fatigue, and exercise intolerance [3], while the two main complications of atherosclerotic cardiovascular disease that cause death are myocardial infarction and ischemic stroke [4]. For decades, the primary therapy for atherosclerotic cardiovascular disease has been statins, which work by lowering plasma cholesterol levels [5,6,7]. However, since cholesterol accumulation precisely occurring in arteries is what drives atherosclerosis [8], statins are only partially effective in treating atherosclerotic cardiovascular disease [9].

The pathogenesis of atherosclerosis is complex and not entirely elucidated [10]. However, factors generally recognized to influence atherogenesis include systemic inflammation, endothelial dysfunction and activation, hypertension, oxidative stress, hypercholesterolemia, altered angiogenesis, oxidized low-density lipoprotein-induced macrophage foam cell formation, and cholesterol efflux [11,12,13,14,15,16,17,18,19,20]. Cholesterol efflux is the process that mammals use to remove excess intracellular cholesterol [20]. Cholesterol efflux is touted as being atheroprotective, since humans do not have the ability to effectively degrade cellular cholesterol [21], and so removing intracellular cholesterol via cholesterol efflux is the process that cells utilize for aiding in the prevention of excess cholesterol accumulation [22,23]. While it is thought that every cell type has the capacity to efflux cholesterol [24], hepatocytes and macrophages are considered to be the major types of cells that participate in cholesterol efflux in mammals [25]. Macrophage cholesterol efflux is acknowledged to be paramount to atheroprotection [26], since impaired macrophage cholesterol efflux is well established as a key driver of atherogenesis, as demonstrated by accelerated plaque development in macrophage-specific ABCA1/ABCG1-deficient mice [27,28]. One notable reason for a possible increase in atherosclerosis from impaired macrophage cholesterol efflux is likely due to reducing reverse cholesterol transport [29], which is the pathway that mammals use to transfer cholesterol from macrophages back to the liver, so that the transported cholesterol may either be excreted or recycled [30,31]. Altered cholesterol efflux in other cells may also contribute to other conditions and disease states, some of which include obesity, type 2 diabetes mellitus, systemic inflammation, and certain forms of cancer [32,33,34,35,36,37,38,39,40,41,42,43,44].

There are four known pathways for macrophage cholesterol efflux [45]. Two transporters that facilitate two of these specific pathways are the ABC transporters, ABCA1 and ABCG1 [46]. It is widely considered that ABCA1 and ABCG1 require cholesterol acceptors to participate in cholesterol efflux [47]. The traditional conceptual model for ABCA1/ABCG1-mediated cholesterol efflux has been that apoAI is the exclusive cholesterol acceptor for ABCA1, while HDL is the exclusive cholesterol acceptor for ABCG1 [48]. However, we and other laboratories have shown that smaller HDL particles also act as cholesterol acceptors for ABCA1 [49,50]. However, while ABCA1 has the capacity to efflux cholesterol to both apoAI and certain HDL particles, macrophage ABCG1 expression may be more important to cholesterol efflux when compared to macrophage ABCA1 expression [51].

Several studies have involved ABCA1 and ABCG1 manipulation in animals [46,52]. However, all of these in vivo models have limitations, especially the animal models that involve attempting to overexpress ABCA1/ABCG1 in certain cells and tissues [49]. In particular, overexpressing ABCA1 and ABCG1 in the macrophages of mice has been extremely challenging, with several drawbacks depending on what method has been used to overexpress the macrophages ABCA1/ABCG1 in vivo [51,53]. Recently, our laboratory characterized a plasmid-based LoxP-Stop-LoxP (LSL) system that permits ABCA1 and ABCG1 overexpression to be induced by the introduction of the enzyme Cre recombinase [49]. Cre recombinase is an enzyme derived from the bacteriophage P1 that recognizes 34bp LoxP sites [54,55,56]. Cre interacting with LoxP sites can lead to site-specific recombination [57,58,59]. This process can be manipulated to delete genes via floxing genes of interest [60], and to overexpress transgenes through the LSL system [49]. The basis of an LSL system involves a "stop" sequence flanked by LoxP sites directly downstream of a promoter and directly upstream from a transgene [61,62,63]. We wanted to utilize this LSL-based strategy in vivo via generating novel mice that harbor ABCA1-LSL or ABCG1-LSL expression cassettes, so that ABC-transporter overexpression can be controlled in certain cells and tissues at a particular timepoint. We isolated and cultured the peritoneal macrophages (PMs) of these mice to determine whether ABC-transporter expression can be induced via Cre. When we successfully introduced Cre to these PMs, we detected a significant increase in ABC-transporter expression and apoAI/HDL-mediated cholesterol efflux. These results show that these newly created mice can be utilized to cross with macrophage-specific Cre mice for macrophage ABCA1/ABCG1 overexpression in vivo through the introduction of Cre recombinase [64]. Furthermore, cross-breeding of these novel mice with various other Cre-driver mice may potentially allow ABC-transporter overexpression to be strictly controlled in several types of cells and tissues by the use of Cre-Lox technology [60,65].

## 2. Materials and Methods

### 2.1. Generation of ABCA1-LSL and ABCG1-LSL Mice

The creation of ABCA1-LSL and ABCG1-LSL mice was conducted at the Medical University of South Carolina Transgenic & Genome Editing Core. Briefly, 20 µg of plasmid DNA containing either ABCA1-LSL or ABCG1-LSL cassettes was subjected to *Not*I digestion to release the respective LSL-based expression cassettes [49]. Along with containing LSL sequences that only allow ABC-transporter overexpression when Cre is present, these expression cassettes also contain the ubiquitous CAG promoter to drive constitutive transgenic expression [49]. The two plasmids containing the LSL-based expression cassettes (pABCA1-LSL and pABCG1-LSL) were constructed by VectorBuilder (Chicago, IL, USA), and the basic plasmid elements of both vectors are a pUC origin or replication (*ori*) and ampicillin resistance gene (Supplementary Figure S1A,B). The purified cassettes were used for pronuclear injection into single-cell C57BL/6 mouse embryos [66]. After parturition, founder mice were initially screened for potentially harboring LSL transgenic alleles by extracting gDNA from tail snips to use for genotyping the mice via end-point PCR. Founder mice that screened positive for hemizygous (^+/0^) ABCA1-LSL or ABCG1-LSL transgenic alleles were shipped to the Clemson University Godley-Snell Research Center vivarium to be bred with C57BL/6 wild-type mice. The progeny of mating pairs were again screened for harboring either hemizygous ABCA1-LSL alleles or hemizygous ABCG1-LSL alleles by extracting gDNA from ear-punches, so that the mice could be genotyped via end-point PCR. To attempt to prevent breeding mice that exclusively harbor the LSL-based expression cassettes within Y chromosomes [67], we selected one specific female ABCA1-LSL^+/0^ offspring and one specific female ABCG1-LSL^+/0^ offspring to use for establishing two separate novel ABCA1-LSL^+/0^ and ABCG1-LSL^+/0^ mouse lines. Once these two mouse lines were established and expanded, we humanely euthanized adult male and female ABCA1-LSL^+/0^ mice and ABCG1-LSL^+/0^ mice through CO_2_ overexposure in a gas chamber followed by cervical dislocation, and we immediately proceeded with isolating the PMs from euthanized mice. All mouse husbandry, breeding, maintenance, and euthanasia performed at Godley-Snell Research Center were approved by Clemson University’s Institutional Animal Care and Use Committee (IACUC).

### 2.2. PM Isolation

We isolated and cultured PMs from ABCA1-LSL^+/0^ mice and ABCG1-LSL^+/0^ mice by adapting a previously described protocol [68]. Briefly, we restrained the limbs of euthanized mice, exposed the peritoneal cavity, and injected the peritoneal cavity with PM isolation medium, which was composed of a final concentration of 3% fetal bovine serum (Avantor Seradigm, Radnor, PA, USA) diluted in sterile phosphate-buffered saline (PBS). After filling the peritoneal cavity with PM isolation medium, we gently massaged the mice’s abdominal wall to dislodge peritoneal cells, so that these cells would become submerged in the PM isolation medium. After removing the skin following peritoneal injections and then gentle massaging, we subsequently collected the peritoneal cells suspended in PM isolation medium in a sterile tube and centrifuged the peritoneal lavage at 500× *g* for 10 min to pellet the peritoneal cells. After removing the PM isolation medium, we resuspended the cell pellet with RBC lysis buffer (Tonbo Biosciences, San Diego, CA, USA), re-centrifuged the cells, and removed the buffer. We then resuspended the cell pellet in PM growth medium consisting of RPMI 1640 medium with L-glutamine (Cytiva, Marlborough, MA, USA), fetal bovine serum (10% final concentration; Avantor Seradigm), and penicillin–streptomycin (1% final concentration; Corning, New York, NY, USA), and then plated the cells in either 48-well tissue culture plates or sterile 4-well chamber tissue culture slides to use for downstream experiments. To maintain PMs in culture, the incubation conditions were set at 5% CO_2_ and 37 °C. Before beginning any treatment or intervention, we first rinsed the cells with PBS to remove any non-adherent cells, cell debris, and dead cells, and then we replenished adherent cells with PM growth medium, so that only PMs likely remained in the treatment plates/chambers to be used in our ex vivo cell culture studies.

### 2.3. PM Characterization

To determine whether PMs were isolated from the peritoneal cavities of mice and these were the cells that survived during the culture conditions described above, we performed immunofluorescent staining to characterize these cells. For these experiments, we initially plated cells in chamber slides and then prepared these cultured cells for staining. Briefly, cells were fixed in 4% paraformaldehyde/PBS, permeabilized in 0.2% Triton-X-100 in PBS, and blocked in 3% bovine serum albumin/10% goat serum/20 mM glycine. The cells were then incubated in either of the following primary antibodies diluted 1:50 in 1% BSA/PBS at 4 °C overnight: anti-CD11b, Invitrogen (Waltham, MA, USA) catalog #14-0112-82; or F4/80, Invitrogen catalog #14-4801-82. Following primary incubation, we incubated the cells with an Alexa Fluor 568 goat anti-rat IgG secondary antibody (Thermo Fisher, Waltham, MA, USA; catalog #A11077) diluted 1:200 in 1% BSA/PBS for one hour at room temperature. Finally, the cells were counterstained with DAPI and mounted in Prolong Gold (Thermo Fisher). Cells were then imaged using a Leica SPE confocal microscope (Leica Microsystems, Buffalo Grove, IL, USA). To view Alexa Fluor 568, cells were excited with a 532 nm laser (emission 580–630 nm, colorized red), and to view DAPI, cells were excited using a 405 nm laser (emission 415–450 nm, colorized blue). Images were collected using a 63X oil lens (N.A. = 1.3) and overlaid and exported using LAS X software (version 3.5.2.18963).

### 2.4. Plasmid DNA Transfection

We used conventional transfection established in our laboratory [69] to attempt to transfect PMs with a GFP-expressing plasmid (System Biosciences, Palo Alto, CA, USA). We also attempted to introduce GFP-expressing plasmids to PMs via utilizing an advanced, non-liposomal polymeric plasmid DNA delivery system acknowledged to be superior for transfecting primary macrophages when compared to conventional plasmid DNA transfection [70,71]. For this alternative plasmid DNA transfection protocol, we adhered to the manufacturer’s directions by first adding pGFP to Opti-MEM™ I Reduced Serum Medium (Gibco, Waltham, MA, USA) contained in a sterile tube. After mixing well with pipetting, we added *Trans*IT-X2 (Mirus Bio, Madison, WI, USA) to this mixture, pipetted to mix thoroughly, and allowed the mixture to incubate at room temperature for 20 min, and then we added this solution drop-wise to cultured PMs. Afterwards, we evenly distributed this solution to the PMs by gently agitating the treatment plates. Mock-transfected PMs were used as control cells. Forty-eight hours after the respective transfection procedures, we imaged PMs for the detection of GFP, utilizing an EVOS 5000 imaging system (Thermo Fisher) and a GFP light cube (ex/em: 470/525 nm).

### 2.5. Adenoviral Infection and Lentiviral Transduction

We used adenovirus and lentivirus to determine whether transgenes could be robustly introduced to PMs using these viral vectors. For adenovirus, we incubated PMs with 10 IFU per cell [72] of a GFP-expressing adenoviral vector (VectorBuilder). For lentivirus, we attempted to transduce PMs with 10 MOI of a GFP-expressing lentiviral vector [73,74] (VectorBuilder). For control PMs, we incubated these cells with the vehicle only instead of a virus. After 48 h of either adenoviral infection or lentiviral transduction, we imaged the PMs to assess the presence of GFP-positive versus GFP-negative cells. Image analysis was performed as previously described for PM plasmid DNA transfections.

### 2.6. Gesicle Treatment

To directly deliver Cre to PMs, we incubated cells with gesicles (Takara, San Jose, CA, USA) that contained both Cre recombinase and CherryPicker™ protein [75,76], with the latter allowing us to assess the gesicle internalization efficiency of PMs. We followed the manufacturer’s instructions for treating PMs with gesicles by initially incubating cells with PM growth medium supplemented with 6 µg/mL of polybrene (Sigma-Aldrich, St. Louis, MO, USA), but omitting penicillin–streptomycin from the polybrene-supplemented PM growth medium. We subsequently added gesicles directly to PMs and incubated cells using standard conditions for 6 h. After this incubation step, we removed this treatment medium and replenished the cells with standard PM growth medium and incubated them in standard conditions for 30 h. Control PMs derived from the same mouse received similar treatment conditions, but the gesicles were instead replaced with vehicle only. After 36 h from first exposing cells to gesicles, we imaged the PMs to assess the delivery rate of gesicles to cells. For this image analysis, we used an EVOS 5000 imaging system and a Texas Red light cube (ex/em: 585/624 nm).

### 2.7. Western Blotting

Forty-eight hours after cultured PMs were initially exposed to gesicles or vehicle only, we rinsed the cells with PBS, and then we harvested the protein [77] from PMs by using radioimmunoprecipitation assay buffer (RIPA) supplemented with protease inhibitors (VWR Life Science, Radnor, PA, USA), with 100 μL of this lysis buffer per well being used for cell lysis. After collecting lysates, we performed centrifugation (10,000× *g* for 10 min) to pellet cell debris, decanted the supernatant, and discarded the cell pellets. We calculated the protein concentrations [78] in PM lysates with a BCA assay (BioVision, Milpitas, CA, USA), and we loaded an equal mass of protein (1 μg) from lysates onto SDS-PAGE gels that were used for protein separation. We then transferred separated proteins onto PVDF membranes (Merck Millipore, Burlington, MA, USA), incubated the PVDF membranes in blocking buffer [79], and then probed for either ABCA1 (1:500 dilution, sc-58219; Santa Cruz Biotechnology, Dallas, TX, USA) or ABCG1 (1:2000 dilution, NB400–132; Novus Biologicals, Littleton, CO, USA). Loading controls for our immunoblots included the housekeeping proteins GAPDH (1:750 dilution, sc-365062; Santa Cruz Biotechnology) and HSP90 (1:2500 dilution, 610419; BD Biosciences, San Jose, CA, USA). After primary antibody incubation, we incubated the PVDF membranes with the following horseradish peroxidase (HRP)-conjugated secondary antibodies: HRP-conjugated goat anti-mouse IgG (1:10,000 dilution, AP181P; Sigma-Aldrich) and HRP-conjugated goat anti-rabbit IgG (1:10,000 dilution, HAF008; Novus Biologicals). After secondary antibody incubation, we incubated the PVDF membranes with Immobilon ECL Ultra Western HRP Substrate (Millipore Sigma, Billerica, MA, USA) and used a ChemiDoc system (Analytik Jena US, Upland, CA, USA) for HRP detection and image analyses [80]. From our immunoblot images, we quantified signals via utilizing ImageJ software version 1.53a (National Institutes of Health, Bethesda, MD, USA), with ABCA1 signals being normalized to HSP90 (loading control) signals, while ABCG1 signals were normalized to GAPDH (loading control) signals.

### 2.8. Cholesterol Efflux

Twenty-four hours after PMs were first treated with either gesicles or vehicle, we rinsed the PMs with PBS, and then cholesterol-loaded the cells for twenty-four hours with [^3^H] cholesterol (1 μCi/mL; PerkinElmer, Waltham, MA, USA) diluted in medium containing BSA-FAFA (2 mg/mL; Sigma-Aldrich) and penicillin–streptomycin [81]. After loading the PMs with cholesterol, we rinsed the cells with PBS and incubated them in efflux medium that contained either vehicle only, apoAI (50 μg/mL; Academy Bio-Medical Company, Houston, TX, USA), or HDL (50 μg/mL; Academy Bio-Medical Company) [74]. Twenty-four hours after exposing the PMs to either cholesterol acceptors or vehicle, we filtered the medium to remove any non-adherent PMs, rinsed adherent PMs with PBS, and collected PM extracts with sodium hydroxide. We utilized a PerkinElmer Tri-Carb 4910TR liquid scintillation counter to measure [^3^H] in filtered medium and PM extracts. We then calculated cholesterol efflux as previously described [82].

### 2.9. Statistical Analyses

We utilized SigmaPlot Software Version 16.0 (Systat Inc., San Jose, CA, USA) to conduct statistical analysis. Normality and equal variance assumptions were analyzed with Shapiro–Wilk tests and Brown–Forsythe tests. When both assumptions were satisfied, we performed a Student’s *t*-test. When normality was not assumed, we performed a Mann–Whitney rank-sum test. When equal variances were violated, we performed Welch’s *t*-test. The level of statistical significance was set at *p* < 0.05.

## 3. Results

### 3.1. Use of Immunofluorescent Staining to Characterize Murine PMs Cultured Ex Vivo

**Figure 1:**
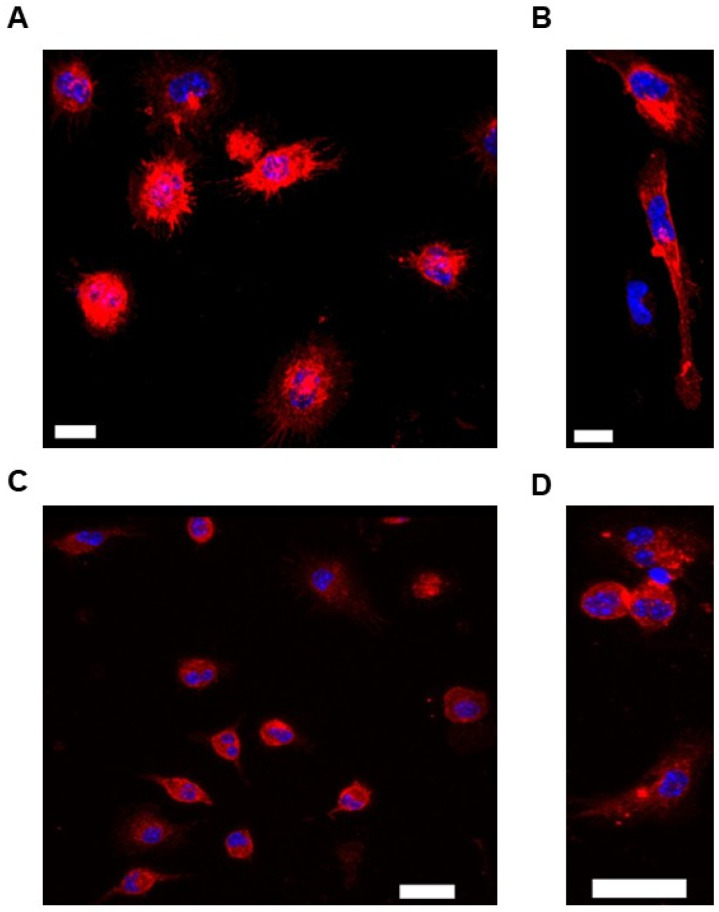
Characterizing cells isolated from the peritoneal cavities of ABCA1-LSL^+/0^ and ABCG1-LSL^+/0^ mice: (**A**–**D**) Representative images show PM staining to detect either CD11b (**A**,**B**) or F4/80 (**C**,**D**). PMs were derived from either a male ABCA1-LSL^+/0^ mouse (**A**,**C**) or female ABCG1-LSL^+/0^ mouse (**B**,**D**). Scale bars shown as 10 µm (**A**,**B**) or 20 µm (**C**,**D**). Cell nuclei were detected with DAPI nuclear counterstain, and images were obtained at 63× magnification (**A**–**D**).

To assess the successful ex vivo isolation of murine PMs, we analyzed the presence of the immune cell marker CD11b [83] and the mature macrophage marker F4/80 [84,85] in cells isolated from the peritoneal cavities of mice that had been maintained in culture. Within these cells, we were able to detect each marker, as determined via immunofluorescent staining (Figure 1A–D). From this result, we concluded that we were successful in the ex vivo isolation of murine PMs and capable of maintaining these cells in cultured conditions.

### 3.2. Plasmid Transfection Is Ineffective at Introducing Transgenes to PMs

**Figure 2:**
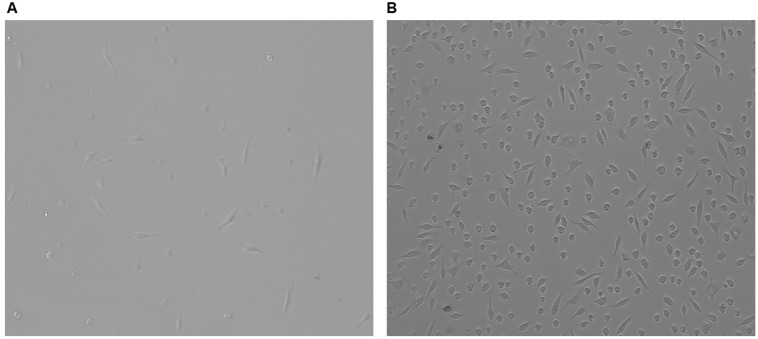
Failure to deliver plasmid DNA into PMs via plasmid transfections: (**A**,**B**) Representative images of attempted pGFP transfection into PMs by using either conventional transfection methods (**A**) or a non-liposomal polymer-based delivery system (**B**).

To permit ABC-transporter overexpression in PMs harboring the LSL-based cassettes, we first needed to efficiently introduce Cre to these cells [49]. Therefore, we first gauged the applicability of utilizing plasmid transfection for potentially introducing Cre to PMs. We assessed the transfection efficiency of both conventional plasmid transfections and a more advanced, non-liposomal polymeric plasmid transfection method [69,70,71] to attempt to deliver GFP-expressing plasmids to PMs. Interestingly, both techniques failed to transfect PMs, based on the absence of GFP^+^ cells observed during cellular imaging (Figure 2A,B). This result implies that plasmid transfection is ineffective in delivering genetic material into PMs.

### 3.3. Adenovirus and Lentiviral Vectors Fail at Robustly Introducing Transgenes to PMs

**Figure 3:**
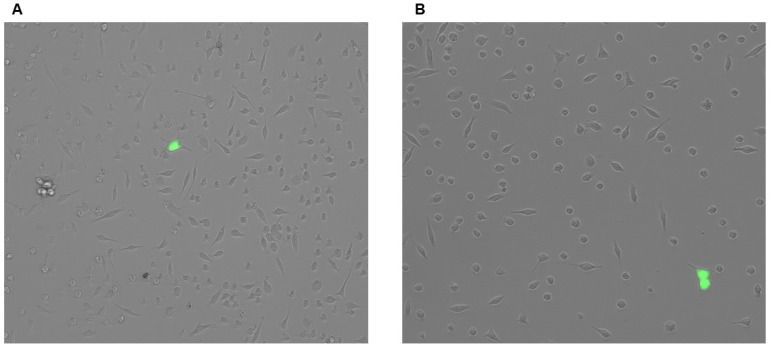
Adenoviral infection and lentiviral transduction are insufficient for cultured PMs: (**A**,**B**). Representative images of PMs incubated with GFP-expressing viral vectors that are either adenovirus (**A**) or lentivirus (**B**).

Since viral vectors are considered to be superior at introducing transgenes to cultured cells when compared to plasmid transfection [86], we next analyzed adenoviral infection efficiency and lentiviral transduction efficiency in PMs to determine whether either of these viruses may be used to effectively deliver Cre to PMs. However, when we incubated PMs with either GFP-expressing adenovirus or GFP-expressing lentivirus, we only detected a sparse population of GFP^+^ cells (Figure 3A,B). From these findings, we concluded that both adenoviral infection and lentiviral transduction of PMs are inefficient at effectively delivering transgenic material to PMs.

### 3.4. PMs Successfully Internalize Gesicles

**Figure 4:**
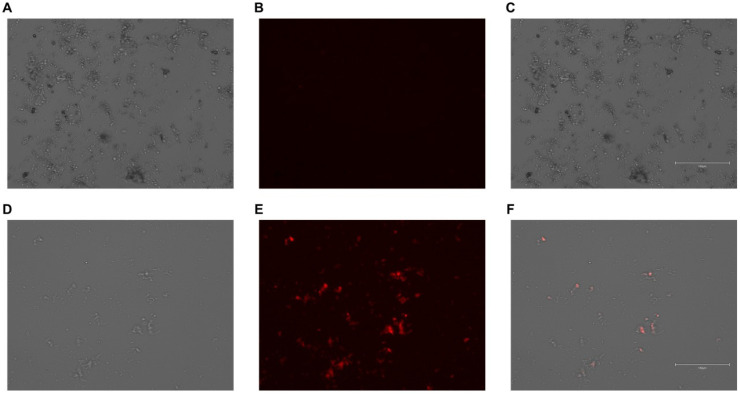
Effective uptake of gesicles by cultured PMs: (**A**–**F**) Representative images of PMs incubated with either vehicle only (**A**–**C**) or gesicles that contain both Cre recombinase and CherryPicker™ protein (**D**–**F**). Images shown are phase-contrast (**A**,**D**), CherryPicker fluorescence (**B**,**E**), and overlay (**C**,**F**). Scale bars shown as 150 µm (**C**,**F**). Images were obtained at 20× magnification (**A**–**F**).

Since we were unsuccessful with using plasmids and viral particles to deliver transgenic material to PMs, we next analyzed whether Cre-loaded gesicles, which are cell-derived nanovesicles [87], may be effectively internalized by PMs. Due to gesicles also containing CherryPicker™ protein [75,76], we imaged PMs incubated with gesicles to assess whether these nanovesicles become internalized by cultured PMs. Our results do show that, upon gesicle exposure, PMs do internalize gesicle particles, based on cellular imaging (Figure 4). From these findings, we infer that gesicles can effectively deliver Cre recombinase to cultured PMs.

### 3.5. ABCA1/apoAI-Mediated Cholesterol Efflux Is Enhanced in ABCA1-LSL^+/0^ PMs Exposed to Gesicles Containing Cre Recombinase

**Figure 5:**
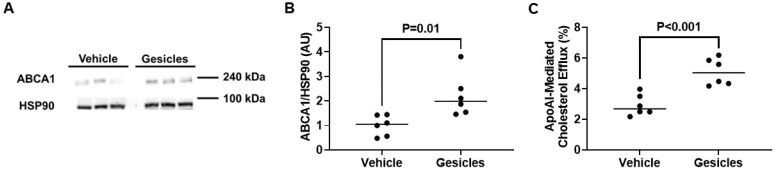
ABCA1-dependent cholesterol efflux is induced in ABCA1-LSL^+/0^ PMs incubated with gesicles loaded with Cre recombinase: (**A**) Representative immunoblot of ABCA1 and HSP90 housekeeping protein for cultured ABCA1-LSL^+/0^ PMs treated with gesicles or vehicle only. (**B**) ABCA1 protein quantification from immunoblot data. (**C**) Cholesterol efflux measured in ABCA1-LSL^+/0^ PMs treated with either gesicles or vehicle only and exposed to apoAI. (**B**,**C**) Data points indicate biological replicates and are derived from PMs isolated from 3 female ABCA1-LSL^+/0^ mice and 3 male ABCA1-LSL^+/0^ mice, with PMs seeded evenly then incubated with vehicle only versus gesicle particles. The uncropped western blot figures were presented in Figure S2.

If delivery of Cre recombinase to cultured ABCA1-LSL^+/0^ PMs is effective, then this would likely result in an increase in ABCA1 protein expression within these cells [49]. Indeed, when we measured ABCA1 expression via immunoblotting in ABCA1-LSL^+/0^ PMs exposed to gesicles containing Cre, we detected a significant increase in ABCA1 protein expression when compared to vehicle-treated ABCA1-LSL^+/0^ PMs (Figure 5A,B; Supplementary Figure S2A,B), which comprised a ~2.6-fold increase in PMs derived from female mice and a ~1.8-fold increase in PMs isolated from male mice. This increase in ABCA1 expression resulted in the ABCA1-LSL^+/0^ PMs incubated with gesicles demonstrating increased apoAI-mediated cholesterol efflux compared to vehicle control ABCA1-LSL^+/0^ PMs (Figure 5C). These changes in cholesterol efflux included ~5.3% (gesicle) versus ~2.5% (vehicle) measured apoAI-mediated cholesterol efflux in PMs derived from female mice and ~4.9% (gesicle) versus ~3.3% (vehicle) measured apoAI-mediated cholesterol efflux in PMs isolated from male mice. Our findings indicate that Cre recombinase stimulates ABCA1/apoAI-mediated cholesterol efflux in cultured ABCA1-LSL^+/0^ PMs.

### 3.6. Delivering Cre Recombinase Gesicles to ABCG1-LSL^+/0^ PMs Increases ABCG1/HDL-Mediated Cholesterol Efflux

**Figure 6:**
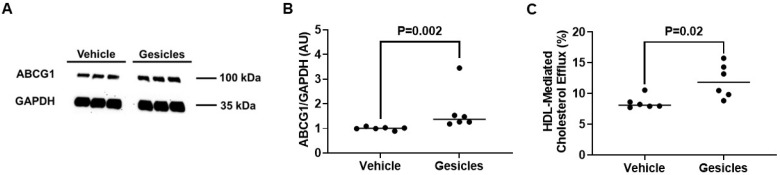
ABCG1-dependent cholesterol efflux is triggered in ABCG1-LSL^+/0^ PMs exposed to gesicles containing Cre: (**A**) Representative immunoblot of ABCG1 and GAPDH housekeeping protein for ABCG1-LSL^+/0^ PMs incubated with gesicles versus vehicle. (**B**) ABCG1 protein quantification from immunoblots. (**C**) Cholesterol efflux assessed in ABCG1-LSL^+/0^ PMs exposed to gesicles or vehicle only and then incubated with HDL. (**B**,**C**) Data points indicate biological replicates and originate from PMs isolated from 3 female ABCG1-LSL^+/0^ mice and 3 male ABCG1-LSL^+/0^ mice, with PMs plated equally and then subjected to treatment with vehicle only or gesicles. The uncropped western blot figures were presented in Figure S3.

Efficient delivery of Cre to ABCG1-LSL^+/0^ PMs would likely lead to increased ABCG1 protein expression [49]. Using immunoblotting, we analyzed the protein expression of ABCG1 within ABCG1-LSL^+/0^ PMs incubated with either vehicle only or gesicles that contained Cre recombinase. In these experiments, we observed a significant increase in ABCG1 protein expression in gesicle-treated ABCG1-LSL^+/0^ PMs versus vehicle control ABCG1-LSL^+/0^ PMs (Figure 6A,B; Supplementary Figure S3A,B), which comprised a ~1.4-fold increase in PMs derived from female mice and a ~2.0-fold increase in PMs isolated from male mice. When we measured HDL-mediated cholesterol efflux in these two groups of treated ABCG1-LSL^+/0^ PMs, we also detected a significant increase in HDL-mediated cholesterol efflux within ABCG1-LSL^+/0^ PMs exposed to gesicles when compared to ABCG1-LSL^+/0^ PMs treated with vehicle only (Figure 6C). These differences in cholesterol efflux included ~11.7% (gesicle) versus ~8.3% (vehicle) measured HDL-mediated cholesterol efflux in PMs derived from female mice and ~12.4% (gesicle) versus ~8.7% (vehicle) measured HDL-mediated cholesterol efflux in PMs isolated from male mice. These results show that Cre has the capacity to induce ABCG1/HDL-mediated cholesterol efflux within cultured ABCG1-LSL^+/0^ PMs.

## 4. Discussion

Our laboratory previously reported how an LSL-based system can control ABC-transporter overexpression through the introduction of Cre recombinase [49]. However, we tested this system in vitro using plasmids that contained the LSL-based expression cassettes [49], which are virtually useless for in vivo applications. Hence, we utilized the previously characterized LSL-based cassettes to create two novel transgenic mouse models: ABCA1-LSL^+/0^ mice and ABCG1-LSL^+/0^ mice. However, before attempting to use these mice in any in vivo experiments, we first wanted to assess whether ABC-transporter overexpression could be induced by Cre using ex vivo approaches. When we delivered Cre recombinase to either ABCA1-LSL^+/0^ PMs or ABCG1-LSL^+/0^ PMs using gesicle particles [87], this did cause ABC-transporter overexpression within these cells, resulting in enhanced apoAI/HDL-mediated cholesterol efflux. However, increased ABC-transporter-dependent cholesterol efflux was negated within PMs that were not incubated with gesicles containing Cre. In summary, our results imply that ABCA1-LSL^+/0^ mice and ABCG1-LSL^+/0^ mice can be implemented in studies where “controlled” macrophage ABC-transporter overexpression is desired. Indeed, use of these mouse models could be applied in future experiments that analyze the impact of macrophage-specific ABC-transporter overexpression on atherosclerosis and reverse cholesterol transport, which may clarify the translational potential of ABCA1/ABCG1 macrophage expression in protecting against atherogenesis.

It has been over 30 years since ABCA1 was successfully identified and cloned using PCR-based approaches [88]. At this time, the function of ABCA1 was not well understood, and it was unknown that a mutation in ABCA1 is what causes Tangier disease, which is a condition that causes cholesterol to become engorged in peripheral cells, accompanied by HDL deficiency [89,90]. However, the chromosomal defect of Tangier disease was later identified [91], followed by studies that confirmed that mutations in the ABCA1 gene are what causes Tangier disease [92,93,94]. Since ABCA1 is involved in both intracellular cholesterol removal and generation of nascent HDL [95], a plethora of publications have since been devoted to studying the potential atheroprotective effects of ABCA1 [96,97,98]. Moreover, a subset of these manuscripts have focused on manipulating macrophage ABCA1 expression in vivo [53,99,100]. However, ABCA1 macrophage overexpression has largely involved bone marrow transplantation from mice that overexpress ABCA1 globally [53]. This approach likely lacks cell-specificity, since this also results in other bone marrow-derived cells besides macrophages exhibiting overexpression of ABCA1, making this strategy a technical limitation that complicates the findings [101]. However, we envision that utilizing ABCA1-LSL^+/0^ mice to overexpress ABCA1 precisely in macrophages by the use of Cre-Lox technology when cross-breeding with macrophage-specific Cre mice could overcome these certain types of limitations.

While ABCG1 was discovered nearly 30 years ago [102,103], there are currently no known diseases or conditions associated with ABCG1 mutations [104,105,106]. However, while ABCG1 is not required for HDL biogenesis per se, ABCG1 does appear to play a large role in impacting HDL size/composition [107,108] and is considered to be the major transporter for HDL-mediated cholesterol efflux [109,110], particularly HDL2 particles [48,50]. Like ABCA1, ABCG1 expression has been studied to analyze this transporter’s impact on atheroprotection [51,111,112,113,114,115,116]. However, strategies previously implemented to overexpress ABCG1 within the macrophages of mice have also involved bone marrow transplantation [112,116], which offers the same drawbacks as using bone marrow-derived cells to overexpress ABCA1 in the macrophages of atherogenic mice, as this technical limitation lacks cell-specificity. However, utilizing Cre-Lox technology via crossing ABCG1-LSL^+/0^ mice with macrophage-specific Cre mice allows ABCG1 to be precisely overexpressed in the macrophages of these mouse models, thereby preventing ABCG1 overexpression from occurring in other types of bone marrow-derived cells besides macrophages.

In our report, we want to highlight some of our shortcomings. One is the failure to deliver transgenes to PMs via transfection. While we note that other labs have been successful with transfecting cultured macrophages with plasmids [117,118], we did not observe successful plasmid transfection, based on the absence of GFP^+^ PMs. We also did not attempt delivering plasmids to PMs via electroporation or nucleofection, which are considered to have superior efficiency compared to transfections when attempting to introduce plasmids to several types of cultured cells [119,120,121,122,123], including macrophages [124,125]. Another limitation is adenoviral infection and lentiviral transduction being inefficient at delivering transgenes to PMs. We acknowledge that other laboratories have been successful using these types of viral vectors to introduce transgenes to cultured macrophages [126,127,128,129,130,131], but these approaches were ineffective for us, as determined by our only observing sparsely populated GFP^+^ cells. However, it is important to mention that other viral vectors may be capable of successfully delivering transgenes to cultured macrophages [132,133,134,135,136] and, thus, may be a more viable option when attempting to introduce transgenes to PMs. Another limitation is our failure to count CD11b^+^ cells, F4/80^+^ cells, and CherryPicker^+^ cells, since cellular imaging is considered to be inferior to flow cytometry for counting viable cells, as well as for accurately identifying cells that are positive for specific markers and reporter proteins [137,138,139]. Hence, selecting cellular imaging over flow cytometry to assess CD11b^+^ cells, F4/80^+^ cells, and CherryPicker^+^ cells can be perceived as a shortcoming of our study. Lastly, our results are only confined to PMs. We selected PMs over other macrophage types (i.e., splenic macrophages and bone marrow-derived macrophages), as murine PMs are considered to be easier to isolate, culture, and maintain ex vivo when compared to other macrophage types [140,141]. While our findings may extend to all types of macrophages within ABCA1-LSL^+/0^ mice and ABCG1-LSL^+/0^ mice, future studies should still be devoted to analyzing whether any differences in ABC-transporter expression are detected in bone marrow-derived macrophages and splenic macrophages when compared to PMs, with and without Cre present, in ex vivo cell culture experiments. Indeed, these findings would be paramount to researchers studying lipid biology and cholesterol metabolism in macrophages, as various macrophage types and subsets exhibit different ABC-transporter expression patterns [105,142,143,144,145].

## 5. Conclusions

In summary, our report shows that PMs from ABCA1-LSL^+/0^ mice are capable of overexpressing ABCA1, and that the PMs from ABCG1-LSL^+/0^ mice have the capacity to overexpress ABCG1, with overexpression being regulated by Cre recombinase activity. While we introduced Cre recombinase to cultured PMs using Cre-loaded gesicle particles, we acknowledge having to use polybrene to facilitate gesicle uptake, which is toxic to macrophages and other cells [131,146,147,148], and so may not be the most appropriate method to introduce Cre when downstream experiments involving analyzing phenotype and assessing cellular function are required. However, using these mice for in vivo studies may potentially provide the opportunity for overexpressing ABC-transporters within the macrophages of mice through Cre-Lox technology. Moreover, use of the tamoxifen-inducible Cre-Lox system may offer additional benefits when compared to more traditional forms of Cre-Lox technology when utilizing ABCA1-LSL^+/0^ mice and ABCG1-LSL^+/0^ mice, as this allows spatiotemporal control of ABCA1/ABCG1 overexpression [149,150]. Indeed, there are several options currently available for macrophage-specific Cre-driver mice [64], including a commercially available tamoxifen-inducible, macrophage-specific Cre mouse model [151]. Regardless of whatever Cre mouse model is used to cross with either LSL-ABCA1 mice or LSL-ABCG1 mice, caution should be exercised concerning potential Cre-mediated activation in off-target tissues if utilizing Cre lines with leaky or broad expression [152]. However, our study only focused on PMs, while hepatocytes also play a pivotal role in atheromodulation, whole-body cholesterol efflux, and reverse cholesterol transport [30,153,154,155]. Thus, future experiments in our laboratory are currently underway to analyze ABCA1/ABCG1-dependent cholesterol efflux within hepatocytes isolated from ABCA1-LSL^+/0^ mice and ABCG1-LSL^+/0^ mice when these cells are introduced to Cre recombinase. Lastly, while we did not examine transgene copy numbers and respective transgenic insertion sites for our two novel mouse models, future directions should involve directly analyzing these features, as this may ultimately impact the findings when incorporating these mice into in vivo studies [156,157].

## Data Availability

All data represented in this work is contained within the manuscript.

## Supplementary Materials

The following supporting information can be downloaded at https://www.mdpi.com/article/10.3390/biology14081073/s1: Figure S1: Plasmid map for pABCA1-LSL (A) and pABCG1-LSL (B). VectorBee was used to generate plasmid vector maps; Figure S2: (A,B) Uncropped immunoblots of ABCA1 (A) and HSP90 (B); Figure S3: (A,B) Uncropped immunoblots of ABCG1 (A) and GADPH (B).
