# Supplementary material for: Ex Vivo Characterization of Peritoneal Macrophages from Novel ABCA1-LSL and ABCG1-LSL Mice for Macrophage-Specific ABC-Transporter Overexpression
Source: Biology (Basel). 2025 Aug 18;14(8):1073. doi: 10.3390/biology14081073 (PMC12383658; doi:10.3390/biology14081073)
Supplement: Supplementary file 1 [file biology-14-01073-s001.zip › biology-3755477-supplementary.pdf]

VectorBee
One Queen to Rule Them All

A

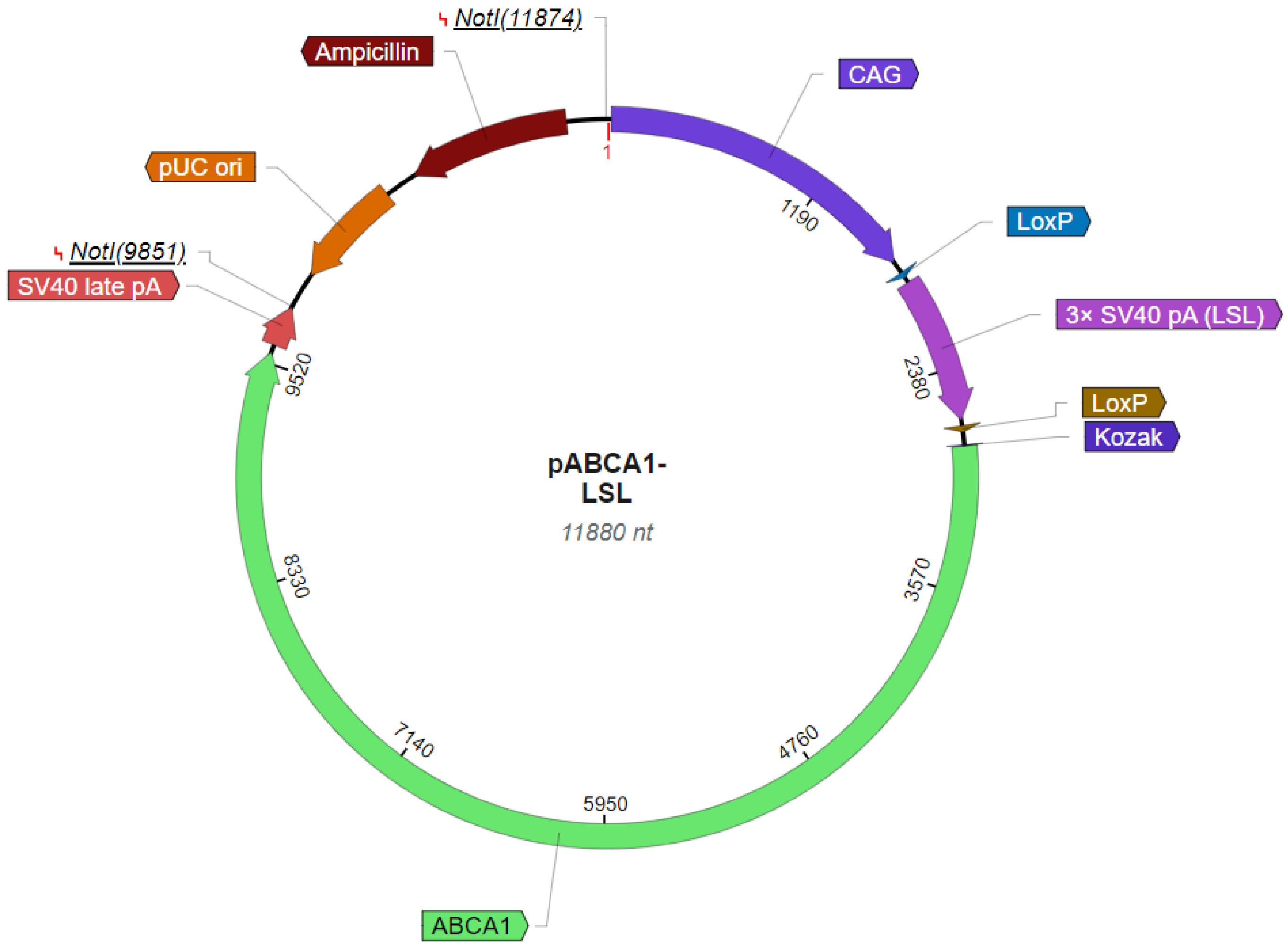

VectorBee
One Queen to Rule Them All

B

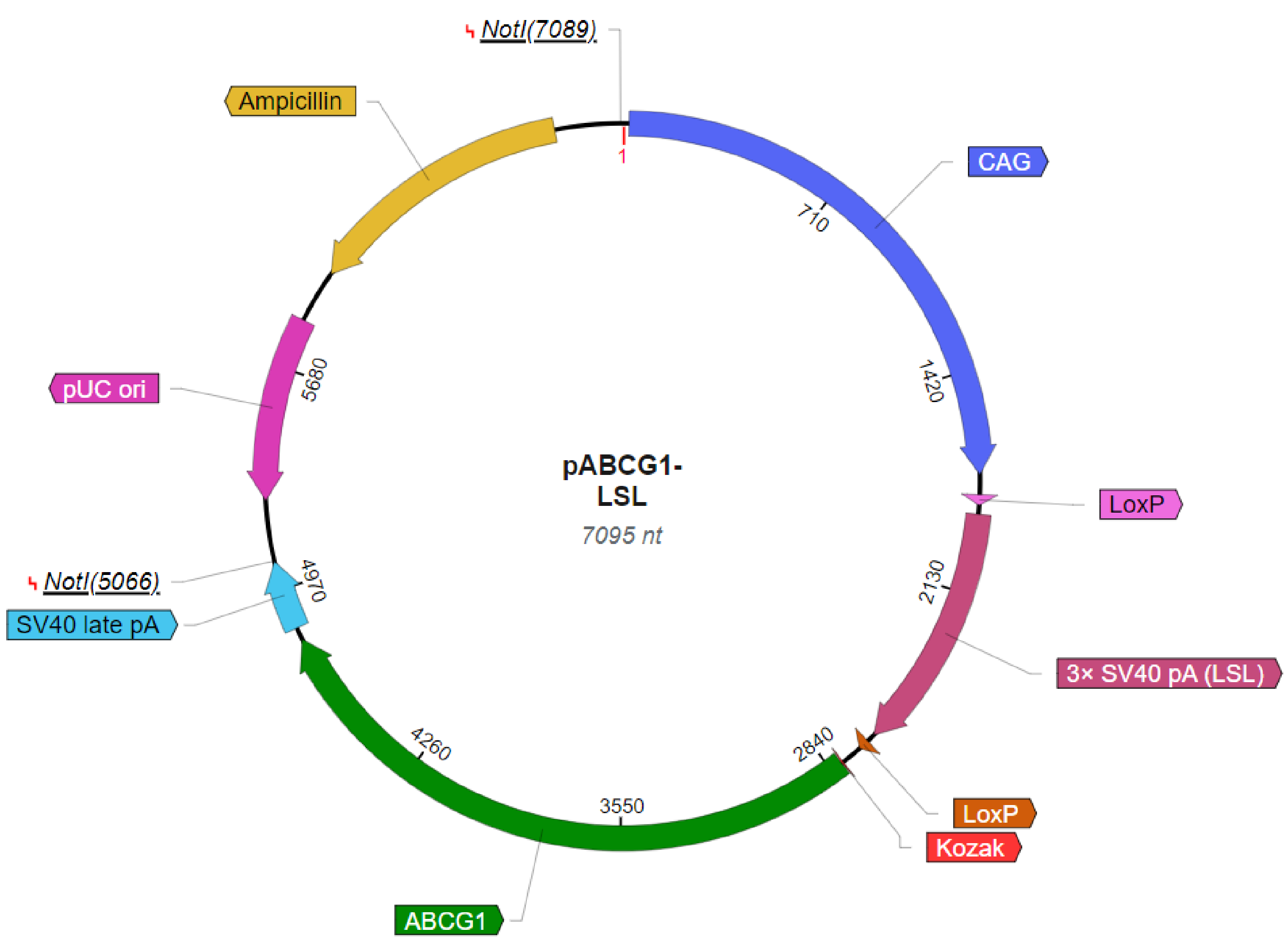

A

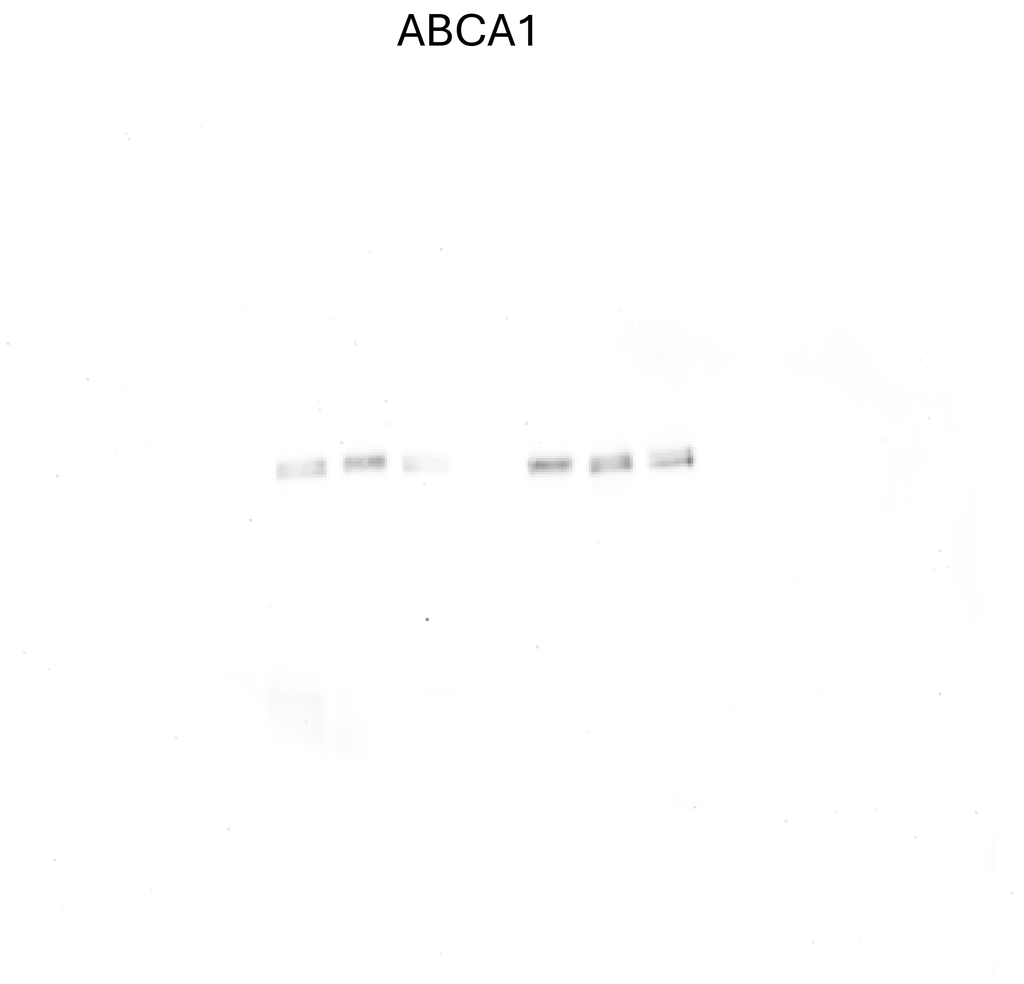

B

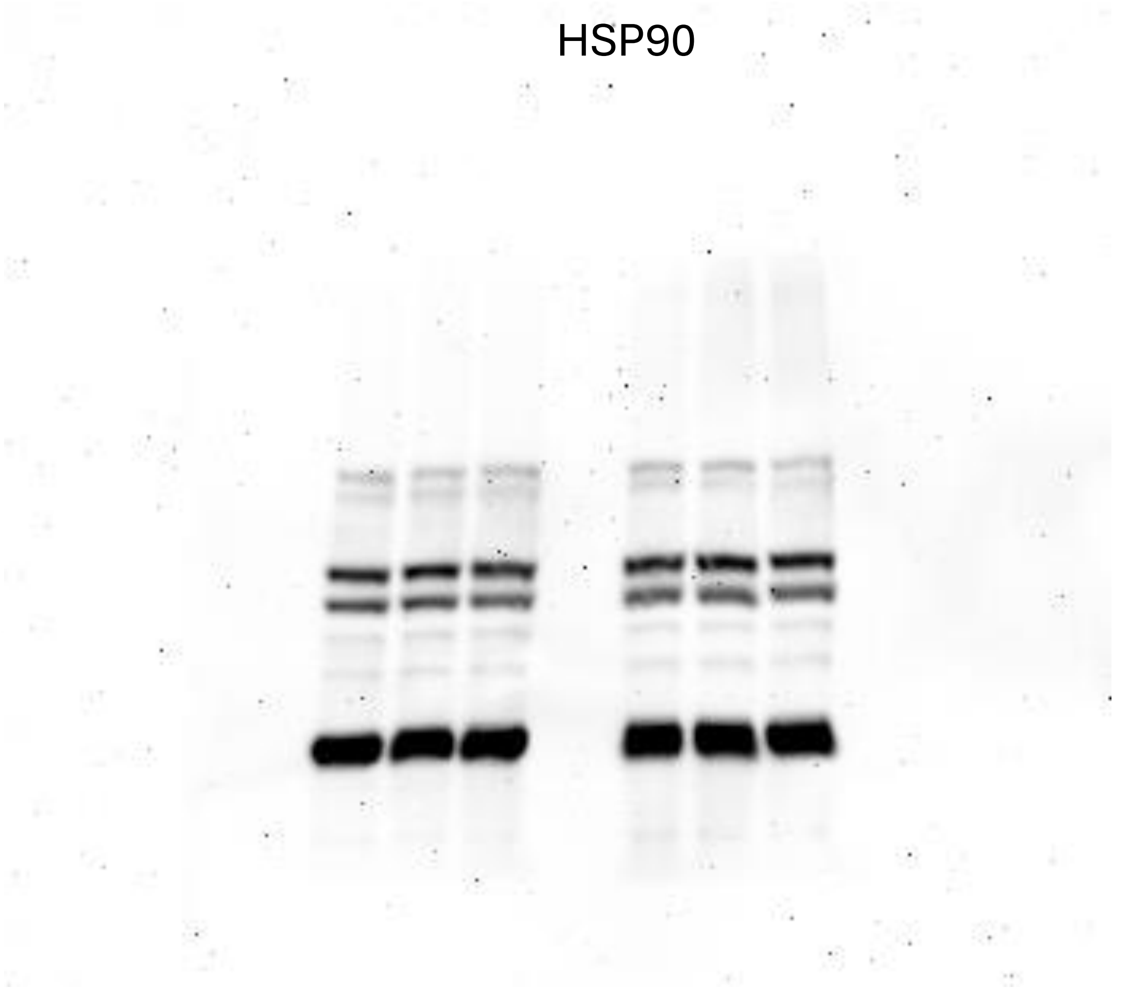

A

ABCG1

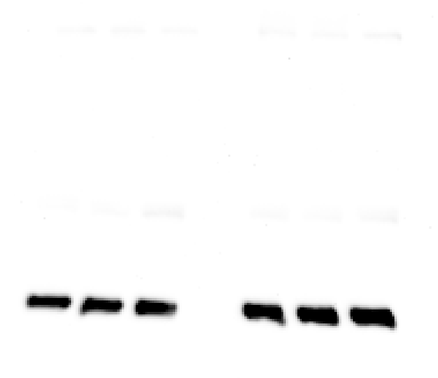

B

GAPDH

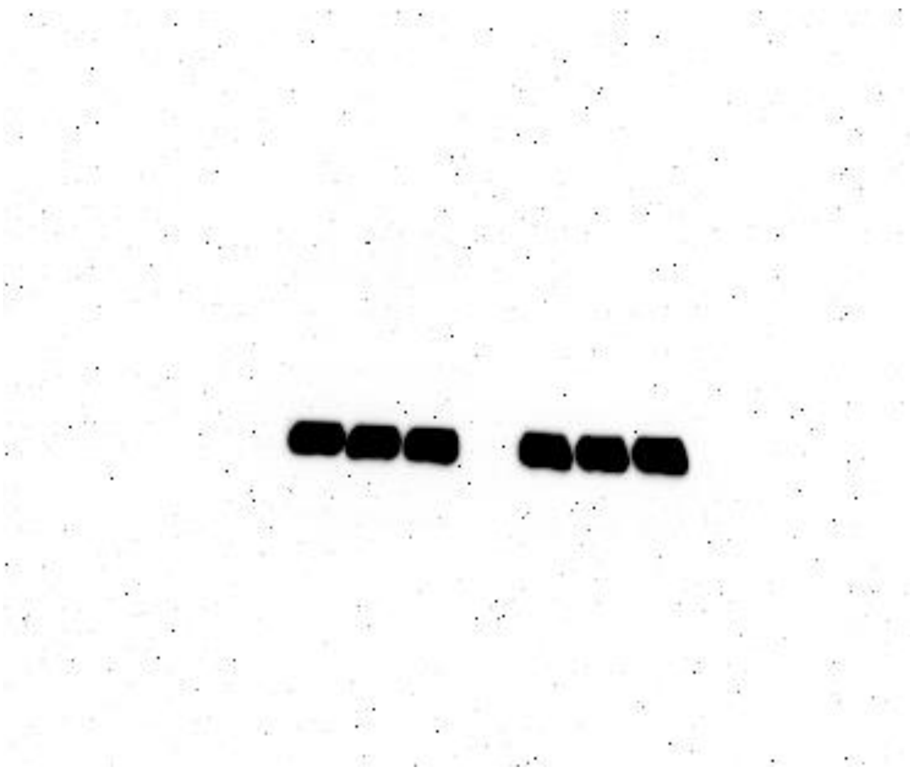

Supplementary Figure Legends

Supplementary Figure S1. A,B) Plasmid map for pABCA1-LSL (A) and pABCG1-LSL (B). VectorBee was used to generate plasmid vector maps.

Supplementary Figure S2. A,B) Uncropped immunoblots of ABCA1 (A) and HSP90 (B).

Supplementary Figure S3. A,B) Uncropped immunoblots of ABCG1 (A) and GADPH (B).
